# Department of Therapeutics

**Published:** 1894-04

**Authors:** 

**Affiliations:** Demonstrator of Physiology and Lecturer on the History of Medicine in the Medical Department of the University of Texas, etc.; Galveston


### Current Medical Literature.

### DEPARTMENT OF THERAPEUTICS.

EDITED BY DAVID CERNA, M. D., PH. D.,

Demonstrator of Physiology and Lecturer on the History of Medicine in the Medical Department of the University of Texas, etc.

LYSOL IN OBSTETRIC PRACTICE.—Israelson (*St. Petersburg Med. Wochen.*, No. 47, 1893) urges the use in obstetric practice of lysol as having greater antiseptic properties and a less poisonous action than any coal-tar derivative hitherto known. He uses 1 per cent. solution for the skin, etc., and a 2 per cent. solution for instruments, this preparation being less milky than the former. No pains, but only slight burning sensations are produced by the use of either, and it has no irritant action on the skin. Owing to lysol forming a "lather" with water, it is a substitute for soap, rendering unnecessary the innunction of the hands, and the application of soap and water prior to operations. The author has tried lysol extensively during one year and a half with satisfactory results.

CONIUM IN TORTICOLLIS.—Wharton Sinkler (*Jour. of Am. Medical Association*, January 20, 1894), of Philadelphia, speaks favorably of the value of conium in the treatment of spasmodic torticollis. He publishes the details of two cases in which the drug rendered marked service, one of them occurring in a lady, 44 years of age, being apparently entirely cured. The author refers to other cases of the same affection, in which conium has done good. Similar satisfactory results were obtained by Sinkler in two or three cases of painless facial spasm, in one of which the spasm entirely disappeared. The author insists in giving the drug in sufficiently large doses. He usually begins with 15 or 20 drops of the fluld extract of the seeds, three times a day, and frequently increases the dose to 60 drops. Though not largely employed in practical medicine, conium seems to possess remedial virtues, and it would appear that, judging from its physiological action, the medicament might be of signal service in the treatment of muscular spasm, particularly of peripheral origin. The above results seem to sustain this idea.

APROPOS OF THE ALLEGED ANTIDOTAL POWERS OF PERMANGANATE OF POTASSIUM IN OPIUM POISONING.—Dr. William Moor, of New York, published an article in the *Medical Record* of February 17, 1894, in which he claimed to have discovered the fact that permanganate of potassium is an antidote to morphine. The results of his experiments were thought to be apparently conclusive (?), so much so, that the mere announcement of them in a daily newspaper of the metropolis produced somewhat of a Koch-like sensation in the over-enthusiastic, unthinking professional minds. With all due respect to Dr. Moor, and admiring his pluck in experimenting upon himself even, we believe, nay, we assert that his results as presented in his paper are *entirely fallacious*, scientifically at least, and calculated to mislead the general practitioner. The permanganate of potassium may be a good antidote to morphine, as has been shown to be so in phosphorous-poisoning by Bodkai, and more recently by Thornton, of Philadelphia. It was found by Bodkai that in the presence of the permanganate of potassium the phosphorus was converted into *ortho-phosphoric acid*, an innocuous substance. Again, Thornton has apparently demonstrated that the permanganate of potassium is a powerful oxidizer of many vegetable alkaloids, and especially so of morphine. Dr. Moor, then, is perhaps on the right track, but the results obtained in such experiments as those performed by himself and of other physicians who have followed him, prove *absolutely nothing!* It is astonishing what large doses of the alkaloid of opium can be given to some of the lower animals, such as rabbits, and particularly dogs, without causing fatal results. This we have repeatedly observed in the laboratory. But as we might be considered too severe in our denunciation of Moor's superficial researches and hasty conclusions, we may be permitted to transcribe part of the contents of a communication on the same subject, and published also in the *Medical Record* of March 10, 1894, by Dr. Glenn Andrews, and with most of which we heartily agree. Dr. Andrews says: "On February 15th, in the presence of Drs. M. L. Wood and J. H. Noftel, of this city (Montgomery, Ala.), I gave a dog weighing ten pounds, three grains of sulphate of morphine by the mouth; in forty-six minutes she had a violent convulsion, attended by a complete paralysis of the hind legs. Two grains of permanganate of potsium were administered, and in fifteen minutes a third grain was given hypodermically. After about twenty or thirty minutes the dog showed but little evidence of the morphia poisoning, except the continued paralysis of the hind legs, which lasted until the following day.

"February 16, two grains of morphine were given to a medium-sized dog, subcutaneously, and in three-quarters of an hour decided symptoms of coma were apparent. One grain of the antidote, hypodermically administered, and followed by a second grain in fifteen minutes, was given. In about half an hour the dog was much improved, and in about three hours was well, except the partial paralysis of the hind legs.

"On February 17, dog No. 1, was given three grains of morphine hypodermically, and suffered much as the previous day. No antidote was given, and the dog recovered completely, though perhaps not so rapidly as before. At the same time dog No. 2 was given six grains of morphine hypodermically. He experienced less sopor, though the general nervous effect seemed more profound than when two grains were administered. After about six hours the dog seemed perfectly well.

"On February 17, in the presence of the Montgomery County Medical Society, I injected hypodermically into a dog weighing fifteen pounds, eight grains of sulphate of morphine. Immediately he showed signs of somnolency followed by excessive nervous excitement and paralysis of hind legs, but in a few hours passed from under the influence of the drug, eating heartily the next day. On the 19th, the same dog was treated to a hypodermic injection of the enormous dose of twenty-four grains of morphine; he suffered much as before, and to-day, the 20th, he is apparently as well as ever, except some continuous nervous excitability. Experiments upon rabbits point to similar results.

"On February 17th, I administered hypodermically one grain of morphine to a small rabbit, and in three hours, noting but little effect other than drowsiness and slowed respirations, the dose was repeated. The animal, without any alarming symptoms, made a perfect recovery in a few hours without antidotes. The same day I gave another rabbit three grains of morphine subcutaneously without lethal effect, and on February 19th, I gave a third rabbit five grains hypodermically, and after a few hours of semi-drowsiness and slowed respiratory action, he made a complete recovery, and is now with the other rabbits and dogs in my barn-yard awaiting another treat of morphine.

"According to the cases referred to by Dr. Moor, the only death occurring was a rabbit given one grain of morphine through the mouth, and subsequently a gastric fistula was established. In my opinion, if the doctor had discovered an antidote for the fistula instead of for morphia, his rabbit would not have died. My experiments have convinced me that one grain of morphine, either introduced through the mouth or hypodermically, will not kill a rabbit, furthermore, five grains subcutaneously will not do so. In dogs, as in rabbits, small doses seem to produce somnolency. However, they bear very large doses of the drug; six, eight, and then twenty-four grains, hypodermically administered, in dogs weighing not over fifteen pounds, have failed in my hands to produce any very alarming symptoms.

"The largest dose reported followed by recovery in dogs, so far as I have been able to find, is six grains given by the stomach. This is surprising to me, since dogs are in such common use for experimental purposes.

"During the past week, in company with several other physicians, I have made a number of experiments bearing on the subject, which I purpose tabulating and forwarding to your valuable journal within the next week. I write this not to critise adversely Dr. Moor's apparent discovery, for, from personal observation I am led to believe that there is something in what he claims, but I wish to suggest to those who may see fit to prosecute these investigations further, to first ascertain the poisonous dose of morphine for the animal experimented upon before publishing an antidote therefor; and, secondly, I wish to note the unusually large doses of morphine which rabbits and dogs will bear with but little inconvenience."

